# Supplementary material for: Time-of-flight detection of terahertz phonon-polariton
Source: Nat Commun. 2024 Mar 13;15:2276. doi: 10.1038/s41467-024-46515-1 (PMC10937925; doi:10.1038/s41467-024-46515-1)
Supplement: Supplementary file 1 — Supplementary Information [file 41467_2024_46515_MOESM1_ESM.pdf]

Supplementary Information for "Time-of-flight detection of terahertz phonon-polariton"

Tianchuang Luo[1,*], Batyr Ilyas[1,*], A. von Hoegen[1,*], Youjin Lee[2], Jaena Park[2], Je-Geun Park[2], and Nuh Gedik[1,✉]

[1]Department of Physics, Massachusetts Institute of Technology, Cambridge, 02139, Massachusetts, USA.

[2]Department of Physics and Astronomy, Seoul National University, Seoul, South Korea.

[✉]e-mail: gedik@mit.edu

February 12, 2024

Supplementary Note 1: Experimental Setup

The schematics of the experimental setup is shown in Fig. S1. Ti:Sapphire based amplifier output is splitted into two arms. The pump arm is sent to an optical parametric amplifier (OPA), whose signal output is set to 1300 nm. THz is generated by nonlinear rectification process by sending 1300 nm beam to an organic crystal BNA and is focused onto the sample with three gold-coated parabolic mirrors. The probe arm is focused on the sample with a lens and the timing between the THz and probe beams is controlled by a delay stage. The reflected second harmonic signal is focused onto a photomultiplier tube (PMT) with another lens, while the reflected fundamental beam is eliminated by a pair of dichroic mirrors and a band pass filter.

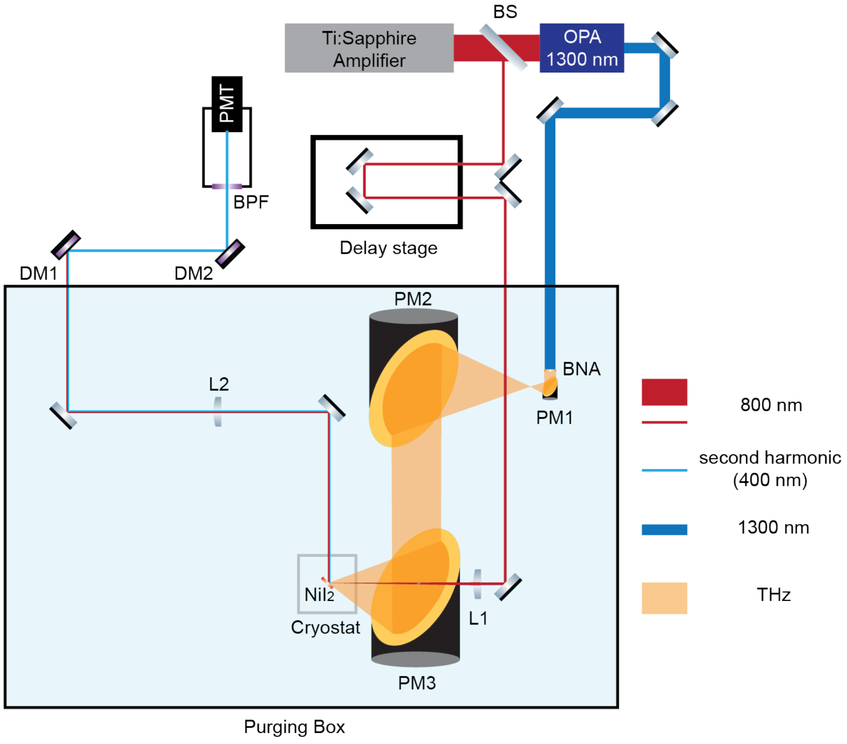

Fig. S1. Schematics of the experimental setup. BS: beam splitter; PM1, PM2, PM3: gold coated parabolic mirrors; L1, L2: lenses; DM1, DM2: dichroic mirrors; BPF: band pass filter.

Supplementary Note 2: THz field induced second harmonic generation and phonon modulation of second harmonic

During the initial few picoseconds of the THz pump SHG probe trace we observe a few sharp spikes and an incoherent background that offsets the oscillation from zero (Fig. 2a). They can be attributed to THz field induced SHG. THz electric field E_{THz} breaks the inversion symmetry and leads to a change in SH intensity:

$$\begin{aligned}\Delta I_{\mathrm{SH}} &\propto |\chi^{(3)} E_{\mathrm{THz}} E^2_{\mathrm{probe}} + \chi^{(2)} E^2_{\mathrm{probe}}|^2 - |\chi^{(2)} E^2_{\mathrm{probe}}|^2 \\ &= \chi^{(3)2} E^2_{\mathrm{THz}} E^4_{\mathrm{probe}} + 2\chi^{(2)}\chi^{(3)} E_{\mathrm{THz}} E^4_{\mathrm{probe}}. \end{aligned} \tag{S1}$$

where $\chi^{(2)}$ and $\chi^{(3)}$ are second and third order nonlinear susceptibility and E_{probe} is the probe electric field. Therefore, we expect ΔI_{SH} components both linear and quadratic to E_{THz}. When the THz pump field is present, the first term in Eq. S1 above results in the incoherent background, and the second term gives rise to spikes near time zero. Moreover, the quadratic term leads to small spectral weight in the high frequency region in the FFT spectrum, which is beyond our THz spectral content. This is due to the fact that quadratic term doubles the frequency.

When the THz field is no longer present, ΔI_{SH} is then dominated by the excited phonon modes. Eq. S1 can be applied in this case by replacing E_{THz} with phonon induced polarization P_{ph}. Since the phonon induced change in $\chi^{(2)} = \chi^{(3)} P_{\mathrm{ph}}$ is much smaller than the static $\chi^{(2)}$ in our experiment, we mainly detect the second term in Eq. S1 which oscillates at the frequency of the phonon mode.

Supplementary Note 3: Comparison between wavelet transform and short-time Fourier transform

Here we compare the wavelet transform (Fig. S2a) and short-time Fourier transform (STFT) (Fig. S2b) of the time trace in Fig. 2a of the main text. The parameters for the wavelet transform is given in the Methods section. The time-window for the STFT is 3 ps. The two analysis give the same behavior of the localized mode and propagating PP and therefore confirm our interpretation. On the other hand, the wavelet transform better resolves the propagating polariton features with less artificial fringes.

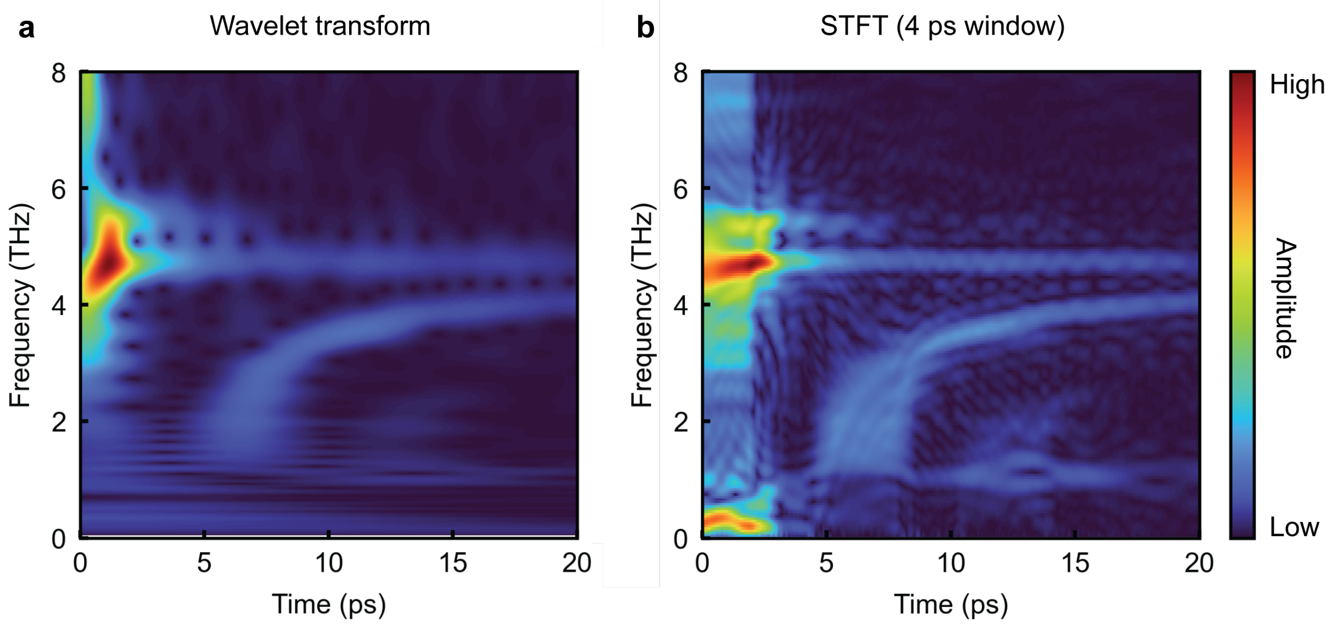

Fig. S2. Wavelet transform (**a**) and STFT (**b**) of the THz induced SH time trace in Fig. 2a.

Supplementary Note 4: THz field strength dependence of the phonon polariton

To confirm the origin of the chirped mode, we studied its dependence on the THz field strength. In Fig. S3a-e we show the spectrogram of the THz pump SHG probe time traces with different THz intensities. The solid white lines show chirped mode frequency as a function of time, and is the same for all figures. The mode frequency as a function of time is not affected by THz fluence, yet the amplitude of the mode decreases with the decrease of THz fluence. In Fig. S3f we show the integrated intensity of the chirped mode along the solid white line in Fig. S3a-e as a function of THz pump electric field. The fitting shows that the mode is driven linearly by the THz pump, as expected for phonon polariton.

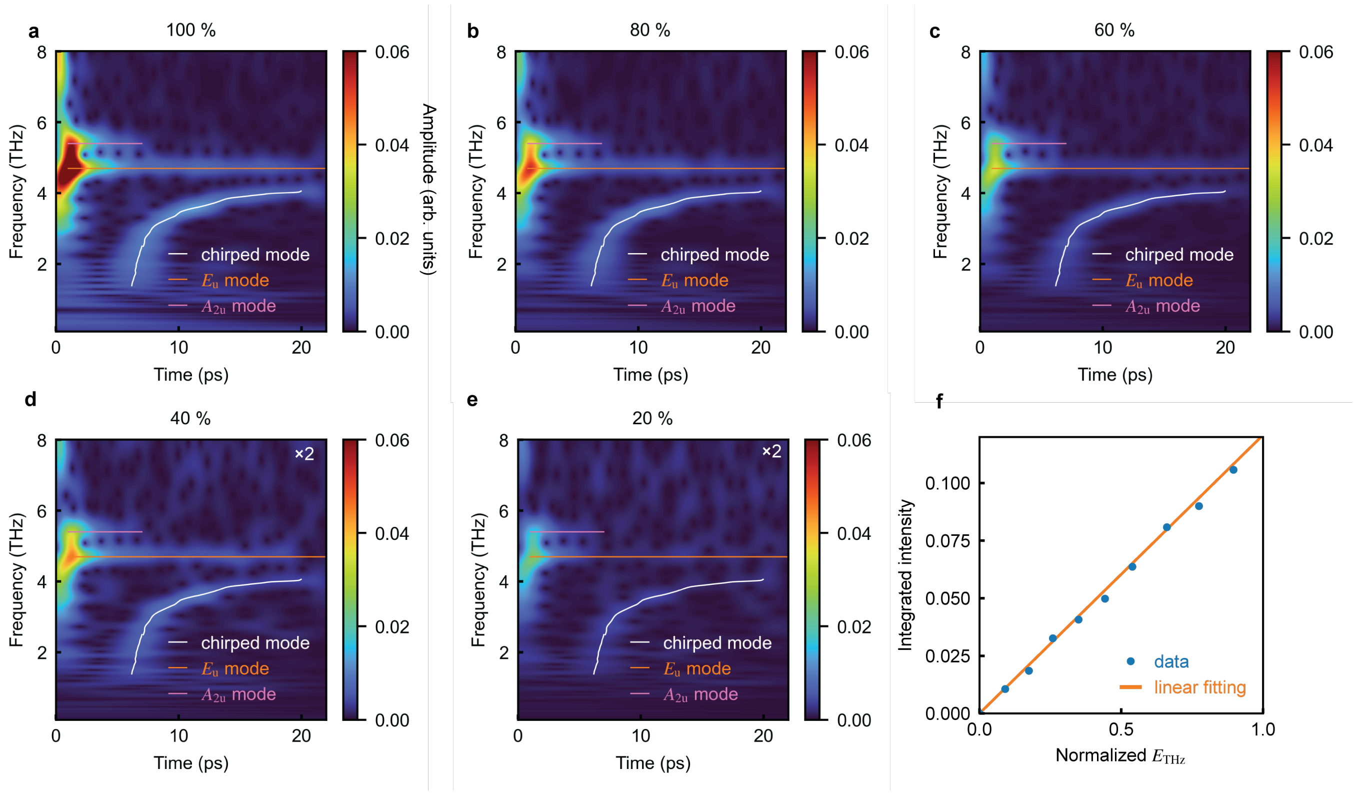

Fig. S3. THz field strength dependence of the phonon polariton. a-e. Phonon polariton observed in spectrogram at different THz field strength. They all fall on to a same $\omega(t)$ relation (white curve). The data in **d** and **e** are multiplied by a factor two to enhance clarity. **f.** The integrated intensity of the phonon polariton along the $\omega(t)$ curve as a function of pump THz field strength. The orange line is a linear fitting to the data.

Supplementary Note 5: Effects of oblique THz incidence angle

The oblique incidence of our THz pulse affects the experimental results in three ways:

1. The propagation direction of the phonon polariton will not be strictly along the out-of-plane direction;
2. The optical distance of the phonon polariton inside the sample will be larger than two times of the sample thickness;
3. The phonon polariton wave packet will have a finite walk-off along the in-plane direction.

To understand how these three factors might affect our results, we first provide an estimation of the angle between the phonon-polariton wavevector and the sample surface normal. The incidence angle of the THz pump is 45°. This angle will be reduced inside the sample due to refraction at the surface. The refractive index of NiI_2 in the relevant spectral range is greater than ~ 3. According to Snell's law, this yields an angle of about 14° between the polariton propagation direction and the surface normal. Therefore, the wavevector can be decomposed as $(0.97e_\perp + 0.24e_\parallel)k_0$, and we probe the dispersion relation along a direction close to the out-of-plane direction. For point 2, the optical distance of the different frequency component of the phonon polariton is given by $d = \frac{2l}{\cos\theta}$. With $\theta < 14°$, d lies between $2l$ and $2.06l$, which only give rise to an uncertainty smaller than 3%, and therefore minimally affects the extraction of polariton dispersion relation. For point 3, the walk-off distance, given by $2l\tan\theta$, is estimated to be smaller than 130 μm, which is within our THz beam spot size. Therefore, the reflected polariton wavepacket will not evade the second harmonic probe as long as the probe and pump spots are spatially overlapping.

To further supplement this semi-quantitative analysis, we re-performed the FDTD simulation with a configuration more closely matching the experimental conditions. In this case we simulate the THz pulse as a Gaussian wave focused on the sample surface, with a full width at half maximum of 180 μm, which is matches the diameter of our THz beam spot. The sample surface normal makes 45° with the THz incidence direction, while its thickness remains the same. The SH response is modeled in the same way as in the main text. The resulting spectrogram is shown in Fig. S4a, which is in qualitative agreement with the simplified one-dimensional (1D) simulation (Fig. S4b and also Fig. 3a in the main text). Quantitatively, Fig. S4c shows the time of the incident and the reflected pulse of the two simulations, where only minimal deviation is observed. The imaginary part of the dispersion extracted from the two simulations are shown in Fig. S4d. The realistic simulation leads to a slight offset of the κ due to the different spatial divergence of PP wavepacket at different frequencies. On the other hand, the realistic simulation does not give rise to any additional peak in $\kappa(\omega)$ as observed in Fig. 4b, which is therefore confirmed to be beyond the single PP behavior.

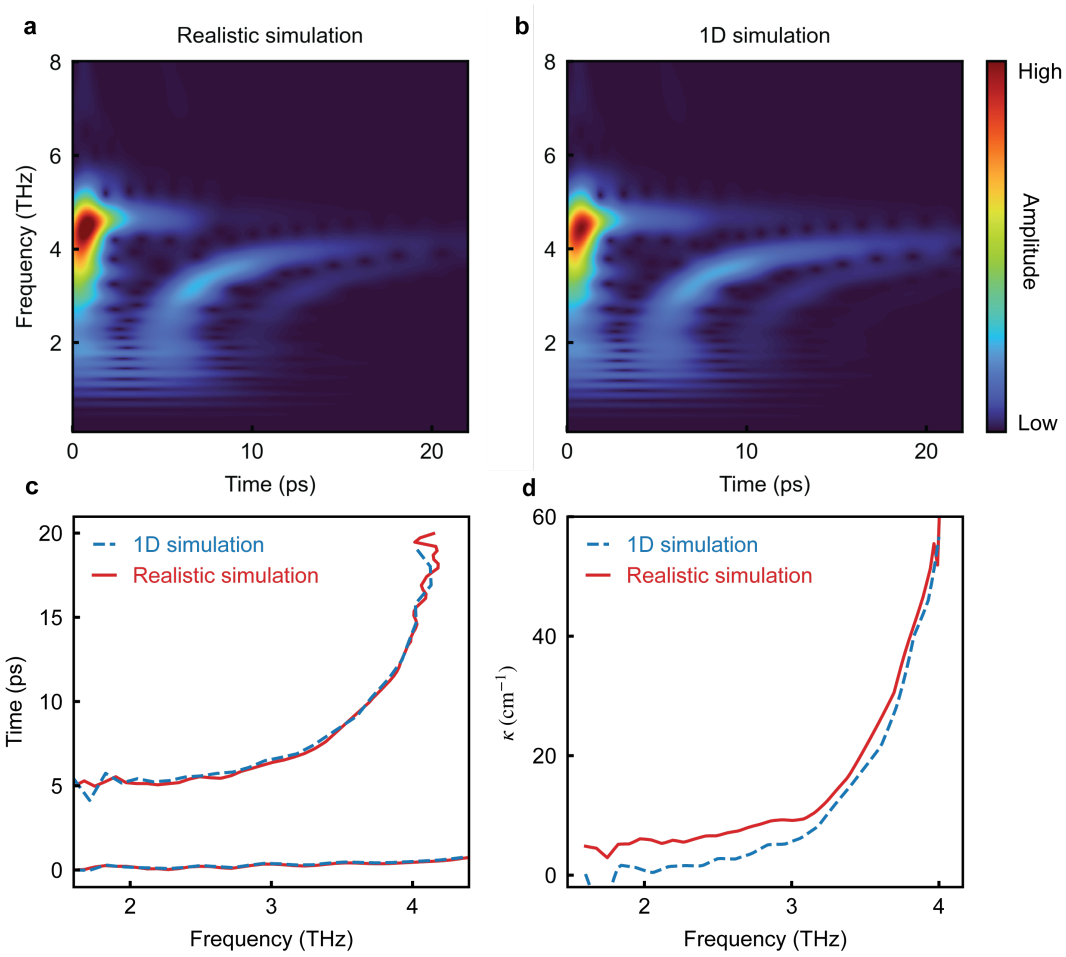

Fig. S4. Comparison of 1D FDTD and more realistic simulations. a-b. Spectrogram from realistic FDTD and simplified 1D simulations. **c.** The arrival time of the incident and the reflected pulses extracted from **a** (red curve) and **b** (blue curve). **d.** The imaginary part of the PP dispersion as extracted from **a** (red curve) and **b** (blue curve).

Supplementary Note 6: Time and frequency resolution from the spectrogram

In this section we analyze the time and frequency resolution in extracting a peak from the spectrogram. The time-frequency resolution is related to the wavelet chosen for the wavelet transform. We chose the generalized Morse

wavelet (GMW) with parameters $\beta = 120$, $\gamma = 3$ as the mother wavelet (see Methods). As can be seen from the representative wavelets in Fig. S5a left and bottom panels, the time-frequency resolution varies with frequency. At the low frequency side of the PP (1.5 THz) the frequency resolution is 0.06 THz and the time resolution is 1.4 ps (Fig. S5a, red wavelets), whereas at the high frequency side, these values are 0.15 THz and 0.5 ps (4 THz) (Fig. S5a, blue wavelets), respectively. The time and frequency uncertainties of the wavelets are shown as the light-shaded region in Fig. S5d. Here the resolution of the wavelet is defined by the square root of its variance.

On the other hand, the temporal step size in our measurements is 0.033 ps, and the measurement time window of 20 ps allows a frequency resolution of 0.05 THz. Because of this oversampling, we can further improve the time/frequency uncertainty by fitting constant frequency/time line cuts with Gaussian functions. We perform the Gaussian fitting along constant-frequency cuts for PP component smaller than 3.5 THz (Fig. S5b) and constant-time cuts for larger than 3.5 THz (Fig. S5c). The uncertainty of this method will be determined by the superposition of the experimental sampling step uncertainty (taken to be half of the experimental time/frequency resolution) and the uncertainty of the peak position obtained by the Gaussian fittings. The final uncertainty window is shown by the solid filled region in Fig. S5d. The time resolution is found to be better than 0.05 ps and the frequency resolution better than 0.03 THz.

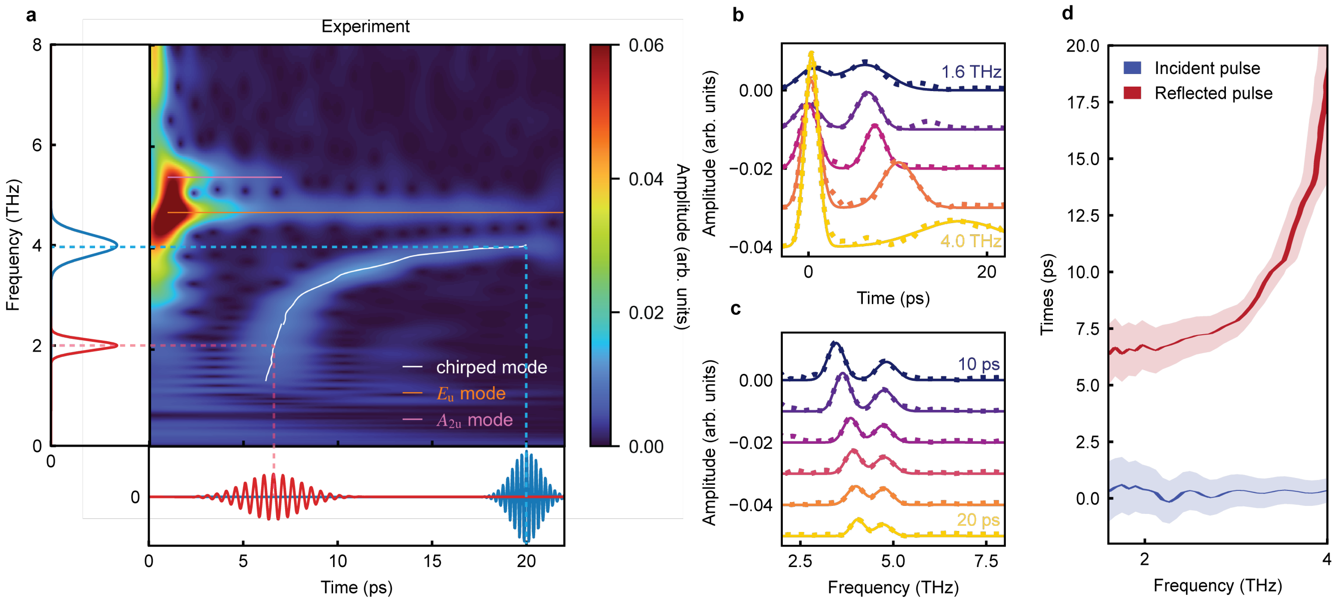

Fig. S5. Time and frequency resolution of the wavelet analysis. a. Wavelet transformed spectrogram aligned with representative wavelet. The real part of the time-domain wavelets are shown in the lower panel, and the frequency-domain wavelet is shown in the left panel. **b.** Constant-frequency linecuts of the spectrogram and and their fittings with two Gaussian peaks. The left (near zero delay) and the right peaks correspond to the incident and the reflected pulses, respectively. **c.** Constant-time linecuts of the spectrogram and their fitting with two Gaussian peaks. The left (lower frequency) and the right peaks correspond to the reflected PP and the localized phonon, respectively. **d.** Frequency-resolved timing of the incident (blue) and the reflected (red) pulses. The light-shaded region shows the wavelet uncertainty window. The solid-shaded region shows our time and frequency resolution.

Supplementary Note 7: PP damping rate at different E_{THz}

To understand the nature of the mode coupling observed in the imaginary part of the dispersion, we extract the PP damping at different E_{THz}. As shown in Fig.S6, the logarithm of the ratio between the reflected and the incident pulse

amplitudes, which is proportional to the imaginary wavevector κ, has a similar ω dependence when E_{THz} is reduced by half. This is an evidence that such mode coupling is independent of E_{THz}, namely in the bi-linear regime.

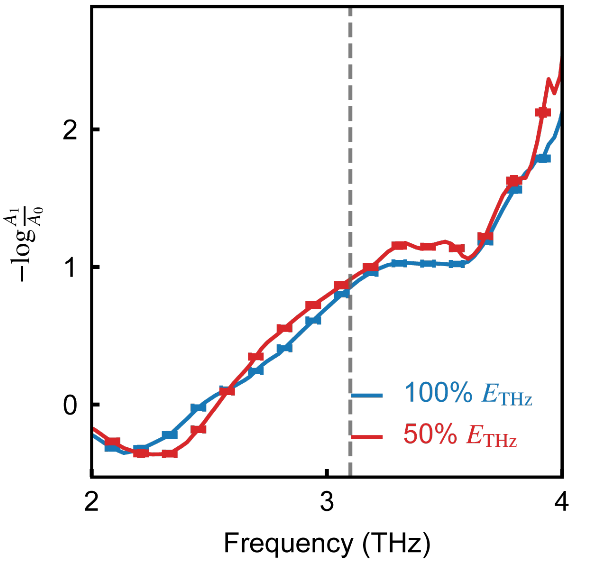

Fig. S6. The logarithm of the ratio between the reflected and the incident pulse amplitudes as a function of frequency at 100% E_{THz} (blue curve) and 50% E_{THz} (red curve). The dashed line shows the frequency of the coupled mode as obtained by bi-linear model fitting in the main text. The error bars are shown at representative points.

Supplementary Note 8: Phonon polariton amplitude obtained from time-frequency analysis

By extracting the SH oscillation amplitude along the white line in Fig. 3a, we can obtain the amplitude of the reflected phonon polariton as a function of frequency. The blue curve in Fig. S7 shows the SH oscillation amplitude normalized by the static SH. The observed SH signal is proportional to the polariton induced electric dipole, which includes contribution from both electrons and phonons (see also in Methods section). When the phonon resonance frequency is approached, the real part of the dielectric function ε_r increases yet the polariton damping also increases, leading to a peak in the polarization amplitude below the resonance frequency (around 3.5 THz in Fig. S7). We can further separate contributions from electronic and phonon polarizations by using the parameters from the fitting in Fig. 3d. The estimated phonon component is shown as the orange line of Fig. S7. The peak of the phonon component is about 0.2% of the static SH signal in the low temperature multiferroic phase.

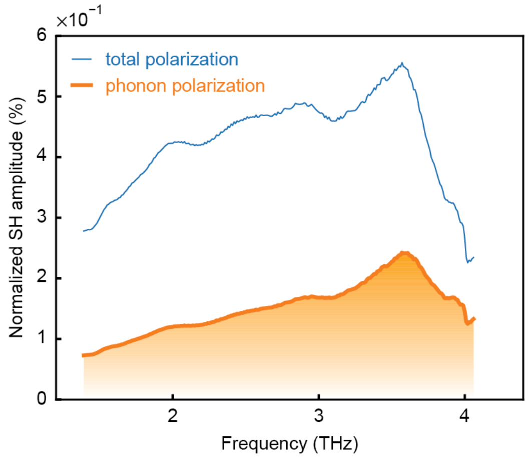

Fig. S7. PP induced electric polarization. The blue curve is the total polarization of the PP returning to the sample surface. The orange curve is the lattice contribution to the polarization.

Supplementary Note 9: Temperature dependence of the phonon polariton

The temperature dependence of THz pump SHG probe spectrogram is shown in Fig.S8. The increase of temperature introduces larger damping to the phonon polariton but have little effect on its frequency.

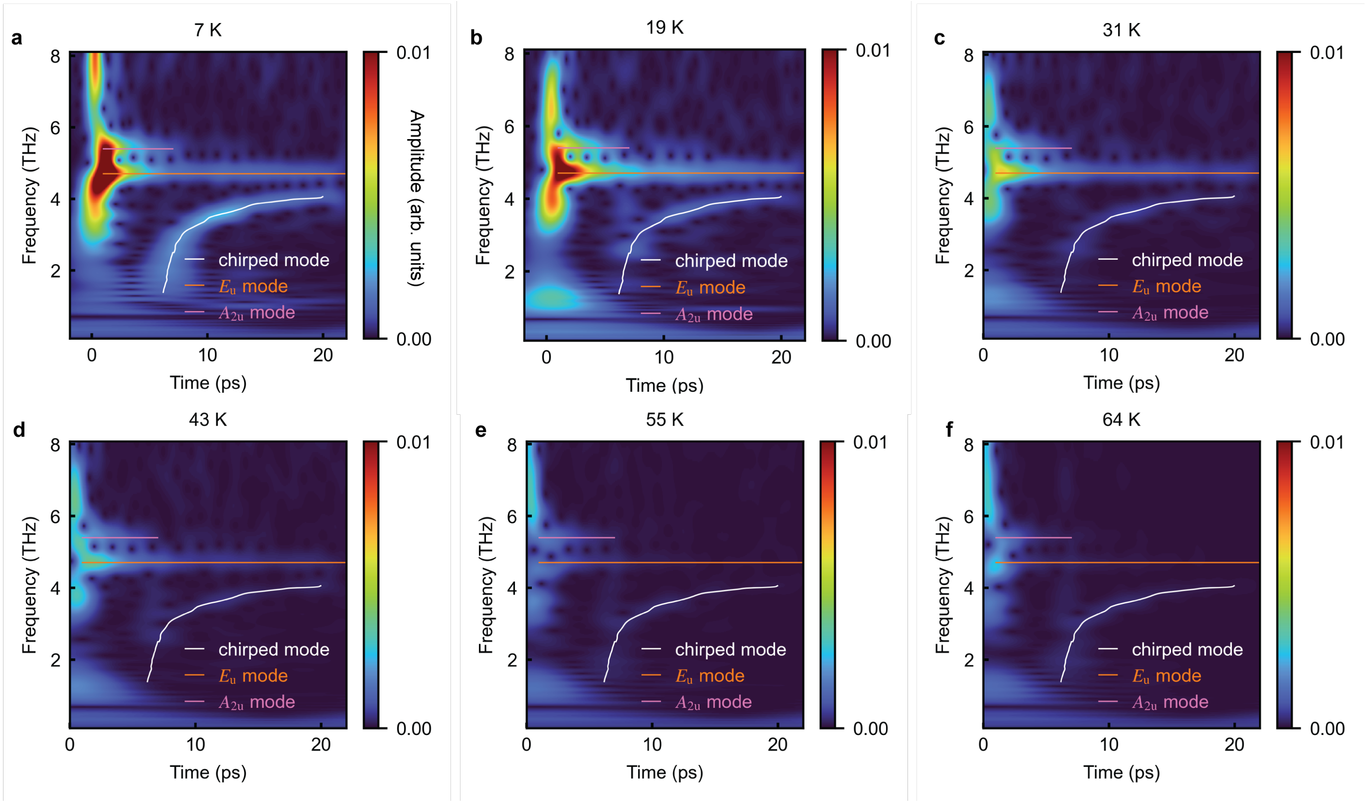

Fig. S8. Temperature dependence of the phonon polariton. a-f. the phonon polariton observed in spectrogram at different temperatures. They all fall on to a same $\omega(t)$ relation (white curve), with smaller amplitude and shorter lifetime at higher temperatures.

Supplementary Note 10: Measuring the upper PP branch with time-of-flight technique

The time-of-flight method developed in our work can capture both lower and upper polariton branches. Any polariton branch that falls in our THz bandwidth can be launched and detected. In NiI_2, however, the upper polariton branch does not fall into our THz bandwidth, and is therefore not detected in our experiments. To demonstrate our sensitivity to the upper polariton branch, we carried out an additional FDTD simulation with an artificially increased THz pump bandwidth. We used the second derivative of a Gaussian peak (Fig. S9a) to create a large bandwidth (Fig. S9b) THz pulse. Upon sending this pulse to the sample, both the lower and upper polariton branches are clearly visible in the wavelet transform (Fig. S9c).

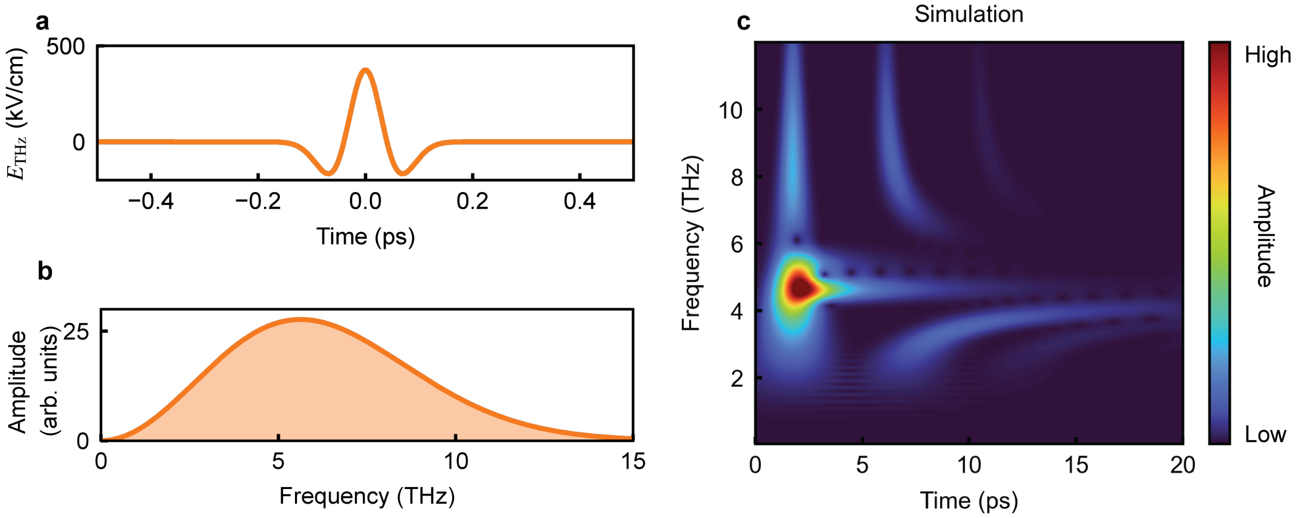

Fig. S9. Simulation of time-of-flight measurement with a large bandwidth THz pulse. a. Time-trace of the THz pulse used for the simulation. **b.** The spectrum of the THz pulse in **a**. **c.** Simulated spectrogram of the time-of-flight measurement with the THz pulse in **a**. Two branches of time-dependent frequency peaks are visible above and below the phonon resonance, corresponding to the upper and the lower PP branch.

Supplementary Note 11: Measuring the dispersion relation of phonon polartion in $MnPS_3$

We applied the same technique to measure the phonon polariton dispersion in another vdW magnet $MnPS_3$. The measurement is performed at 12 K where $MnPS_3$ is in the antiferromagnetic phase. Fig. S10a shows the THz pump SHG probe time trace and Fig. S10b shows the spectrogram. In the spectrogram, a similar chirped mode is visible, as labeled by the solid white line in Fig. S10b. In addition, a second, less pronounced chirped mode is present in the same frequency range, which can be interpreted as phonon polariton travelling two round trips inside the sample. The time frequency dependence of the second chirped mode is well matched by multiplying the time axis of the first chirped mode by two (dashed white line in Fig. S10). The solid orange line in Fig. S10b is another chirped mode observed at higher frequencies, which can be interpreted as the upper branch of the phonon polariton. The upper branch is not fully resolved due to the limitation of our THz bandwidth.

By performing the same analysis as in NiI_2, we integrate $t(\omega)$ and obtain the real part of the PP dispersion relation. The two integrated branches can be fit by the Lorentz model of one PP very well. The fitting yields $\omega_{\mathrm{T}} = 4.78$ THz

and $\omega_L = 5.04$ THz. Although direct measurements of infrared phonon spectrum on $MnPS_3$ is lacking, the fitted ω_T matches Raman spectra[1], as the phonon mode is both IR and Raman active in such an inversion symmetry breaking system. Due to pump probe misalignment on this $MnPS_3$ sample, the analysis on the imaginary part of the dispersion relation cannot be carried out.

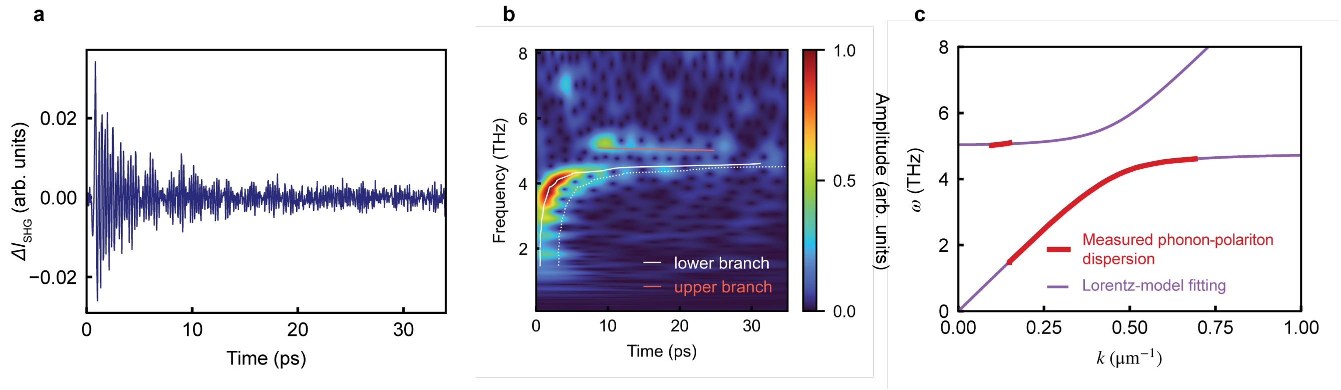

Fig. S10. Phonon polariton observed in $MnPS_3$ **a.** THz pump SHG probe trace on $MnPS_3$. **b.** Spectrogram of the trace in **a**. **c.** the reconstructed phonon polariton dispersion relation from the spectrogram in **b**.

References

1. Kim, K. *et al.* Antiferromagnetic ordering in van der Waals 2D magnetic material MnPS3 probed by Raman spectroscopy. *2D Mater.* **6,** 041001 (2019).
